# Effects of Omega-3 Fatty Acids on Postoperative Inflammatory Response: A Systematic Review and Meta-Analysis

**DOI:** 10.3390/nu15153414

**Published:** 2023-07-31

**Authors:** Ghaith Mohsen, Annika Stroemer, Andreas Mayr, Andrea Kunsorg, Christian Stoppe, Maria Wittmann, Markus Velten

**Affiliations:** 1 Department of Anesthesiology and Intensive Care Medicine, University Hospital Bonn, 53127 Bonn, Germany; andrea.kunsorg@ukbonn.de (A.K.); maria.wittmann@ukbonn.de (M.W.); markus.velten@ukbonn.de (M.V.); 2 Department of Medical Biometry, Informatics and Epidemiology, University Hospital Bonn, 53127 Bonn, Germany; stroemer@imbie.uni-bonn.de (A.S.); amayr@uni-bonn.de (A.M.); 3 Department of Anesthesiology and Intensive Care Medicine, University Hospital Wuerzburg, 97080 Würzburg, Germany; christian.stoppe@gmail.com; 4 Department of Cardiac Anesthesiology and Intensive Care Medicine, German Heart Center Berlin, Charité Berlin, 13353 Berlin, Germany

**Keywords:** perioperative nutrition, preoperative nutrition, omega-3, fish oil, fatty acids, abdominal surgery

## Abstract

Initial evidence indicates that preoperatively initiated administration of omega-3 fatty acids (FAs) attenuates the postoperative inflammatory reaction. The effects of immunonutrition containing omega-3 FAs, such as eicosapentaenoic acid (EPA) and docosahexaenoic acid (DHA), on the inflammatory response to abdominal surgery continues to be unclear, although improved outcomes have been reported. Therefore, we determined the effectiveness of preoperatively initiated omega-3 FAs administration on postoperative inflammation defined as CRP (C-Reactive Protein), IL-6 (Interleukin 6), and WBC (White Blood Count) and potential effects on postoperative length of hospital stay (LOS) due to an improved inflammatory response. Methods: a literature search of Cochrane Library was conducted to identify all randomized controlled trials (RCTs) investigating the effects of preoperatively initiated omega-3 to standard care, placebo, or other immunonutrients excluding omega-3 FAs in patients undergoing abdominal surgery until the end of December 2022. Results: a total of 296 articles were found during the initial search. Thirteen RCTs involving 950 patients were identified that met the search criteria. These were successively analyzed and included in this meta-analysis. There was no significant difference between the groups with respect to inflammatory markers IL-6: −0.55 [−1.22; 0.12] *p* = 0.10, CRP: −0.14 [−0.67; 0.40] *p* = 0.55, WBC: −0.58 [−3.05; 1.89] *p =* 0.42, or hospital stay −0.5 [−1.43; 0.41] *p* = 0.2. Conclusion: although reduced inflammatory markers were observed, preoperative administration of omega-3 FAs immunonutrients had no significant effect on the postoperative inflammatory response in patients undergoing abdominal surgeries. Yet, results obtained from this study are inconclusive, likely attributed to the limited number of trials and patients included. Further studies are required to obtain a better educated verdict.

## 1. Introduction

Surgical trauma elicits a cascade of events resulting in an immune response with activation of the cytokine cascade in the postoperative period. Cytokines play a crucial role in orchestrating the inflammatory response at the site of injury, thereby facilitating the process of wound healing. However, an overabundant production of cytokines can have systemic ramifications leading to postsurgical complications and mortality [1].

Major abdominal surgeries precipitate a systemic inflammatory response, potentially resulting in a risk of serious complications, including organ damage and failure [2].

Several studies were able to show that postoperative inflammatory markers have a prognostic power for postoperative complications and mortality [3,4,5]. Szczepanik and colleagues examined IL-6 serum levels in the post operative period and found that a higher level on the first postoperative day was an independent prognostic factor for postoperative complications [3].

Satonocito et al. evaluated C-Reactive Protein (CRP) levels and kinetics after major surgery (cardiac, neuro, vascular, thoracic, or abdominal) during the postoperative course in about 150 patients and concluded that the patients, who experienced postoperative complications, exhibited higher CRP levels at baseline [6].

It is conceivable to speculate that the reduction of these postoperative inflammatory markers through anti-inflammatory mechanisms could yield favorable outcomes.

Polyunsaturated fatty acids (PUFAs) are critical components of cell membranes as well as precursors of prostaglandins, thromboxanes, and leukotrienes (LTs), playing a crucial role in maintaining fluidity of membrane phospholipids and regulating myriad of cell and tissue responses [7,8,9].

PUFAs are divided into three major groups: omega-3 PUFAs, omega-6 PUFAs, and omega-9 PUFAs. The omega-3 PUFAs mainly include α-linolenic acid (ALA), eicosapentaenoic acid (EPA), and docosahexaenoic acid (DHA), and have been recognized as important components of immune-modulating nutrients [10]. For the last couple of decades, omega-3 FAs have been of interest in the context of inflammatory conditions, and recent meta-analyses have proved their potential ability to reduce chronic inflammation in diseases such as coronary artery disease and diabetes [11,12].

Several studies in rodents and humans [13,14,15] have shown that omega-3 FAs have an anti-inflammatory effect owing to their ability to inhibit the endotoxin-induced production of interleukin-6 (IL-6) by venous endothelial cells. A 2012 study conducted by Kiecolt-Glaser et al. established that the administration of omega-3 fatty acid supplementation was associated with a significant reduction in serum IL-6 levels by as much as 12%, thereby mitigating inflammation in overweight, sedentary, middle-aged adults [16]. Furthermore, a systematic review and meta-analysis by Natto and colleagues from 2019 established that omega-3 fatty acid supplementation was associated with lower levels of inflammatory markers in individuals with diabetes and cardiovascular diseases [12]. A more recent study conducted by our group demonstrated that the administration of docosahexaenoic acid (DHA) resulted in the attenuation of the inflammatory response after an induced myocardial infarction in a closed-chest model leading to a reduction in infarction size in mice [17]. 

Studies have demonstrated that EPA and DHA can suppress the production of eicosanoids derived from arachidonic acid (AA), which are important mediators and regulators of inflammation [18,19]. EPA appears to significantly curb inflammatory reactions, effectively competing with AA. The EPA/AA ratio might potentially be linked to several mechanisms in triggering inflammation, which might lead to cancer development and chronic inflammatory conditions including atherosclerosis and cardiovascular disease [20,21,22].

Given the demonstrated anti-inflammatory effects of omega-3 fatty acids, particularly their ability to decrease inflammatory markers as described in earlier studies, it becomes highly pertinent to investigate their potential benefits within a surgical context, more specifically in abdominal surgeries. 

The primary mechanism through which DHA and EPA present their anti-inflammatory effects is by lowering the production of eicosanoids derived from arachidonic acid (AA). They accomplish this by competing with AA for integration into cell membrane phospholipids, a phenomenon which, in turn, reduces the quantity of AA within these membranes. This reduction is partly attributable to the inhibition of COX-2 (cyclooxygenase-2) and 5-LOX (5-Lipoxygenase) enzymes that act on AA by competing with AA in its metabolism through COX and LOX enzymes [23,24]. DHA and EPA are also associated with a reduced activation of the proinflammatory transcription factor NF-κB (nuclear factor k-light-chain-enhancer of activated B cells) in response to inflammatory triggers. This happens due to the prevention of phosphorylation I-κB, the inhibitory subunit of NF-Κb [24,25].

Furthermore, EPA and DHA lead to the production of Resolvin E and D, respectively, through the COX and LOX pathways. Resolvins have anti-inflammatory and inflammation-resolving properties. They inhibit the transendothelial migration of neutrophils, thus precluding their infiltration into areas of inflammation and inhibiting interleukin-1β (IL-1β) production [23,26]. 

However, the anti-inflammatory effects of omega-3 FAs after major abdominal surgery have yet to be conclusively elucidated. Therefore, we conducted a systematic review and meta-analysis of randomized controlled trials published until December 2022 in patients undergoing major abdominal surgery to provide a robust overview of the anti-inflammatory qualities of omega-3 PUFAs on postoperative inflammation.

## 2. Materials and Methods

In accordance with the PRISMA (Preferred Reporting Items for Systematic Reviews and Meta-Analyses) guidelines, we performed a comprehensive, systematic literature search of the Cochrane Library database for all RCTs examining the effects of perioperative-initiated immunonutrition administration containing omega-3 FAs.

### 2.1. Search Strategy

We searched the Cochrane Database for all RCTs examining the effect of perioperative immunonutrition in patients undergoing major abdominal surgery, using Medical Subject Headings and Emtree terminology to select the studies. 

The following search terms were used: abdominal surgery, perioperative care, surgical procedures, operative, postoperative complications, postoperative care, fatty acids, omega-3, eicosapentaenoic acid, fish oils, fatty acids, unsaturated, docosahexaenoic acids. The literature search was limited to articles published between January 1995 and December 2022.

### 2.2. Inclusion and Exclusion Criteria

RCTs that met the following criteria were included in the meta-analysis: (1) articles written in English; (2) articles that involved adults > 18 years of age; (3) articles that included patients who were scheduled to receive an elective abdominal surgery; (4) articles that examined pre- or pre- and postoperative omega-3-immunonutrition; (5) articles with full text availability.

Exclusion criteria were as follows: (1) immunonutrition given only postoperatively; (2) transplantation surgery; (3) studies that did not publish IL6/CRP/WBC data in any shape or form.

### 2.3. Intervention and Control

The intervention group was administered nutrition enriched with omega-3 fatty acids, while the control group was subjected to either a conventional therapeutic approach (devoid of nutritional supplementation) or an immunonutrition regimen excluding omega-3 fatty acids.

### 2.4. Outcomes

The primary objective of this meta-analysis was to evaluate the postoperative inflammatory response, defined as IL-6, CRP, or WBC within the first 3 postoperative days. The secondary outcome was to assess the length of hospital stay.

### 2.5. Data Extraction

One author (GM) screened the results of the initial searches twice, read the results of the eligible studies, and extracted the data. The results were screened and evaluated by a second author (MV) independently. Discrepancies were discussed and solved jointly. Extracted data included title, first author, sample size, age, sex, type of intervention, type of control, time of initiation of immunonutrition, duration of immunonutrition, IL6/CRP/WBC values. The authors of studies that did not include the mean values of investigated laboratory parameters were contacted and the relevant information was requested. In the instance of failure to receive an answer to our query, data were extracted from published graphs using WebPlotDigitizer V 4. 5 (Ankit Rohatgi Website: https://automeris.io/WebPlotDigitizer, last accessed on 30 September 2022).

### 2.6. Subgroup Analysis

Due to the lack of data, a planned subgroup analysis was not conducted.

### 2.7. Statistical Analysis

We performed a meta-analysis for postoperative inflammatory responses including IL-6, CRP, and WBC and evaluated length of hospital stay (LOS) as a secondary endpoint. Inflammatory responses and LOS were analyzed using random-effect models, with the standardized mean difference assessed as effect size. A random effects model was chosen to account for heterogeneity of disease and the surgical procedure of the included abdominal surgical trials as well as dosage [27]. The DerSimonian and Laird method were used to estimate the cross-study variance and further adjustment by the Hartung-Knapp method for a refined variance estimator for random effect meta-analysis was applied [28,29]. Heterogeneity was assessed using the *I*^2^ statistics and a chi-square test. All analysis were conducted using R version 4.0.4 (15 February 2021), using the “meta” package (version 5.2-0) to perform the meta-analysis and generate the forest plots of the random effects meta-analysis showing the estimated treatment effect and confidence interval of each individual study, as well as the overall effect estimate from all studies.

We considered a *p* value < 0.05 as statistically significant and a *p* value < 0.1 as statistical trend.

## 3. Results

**Figure 1:**
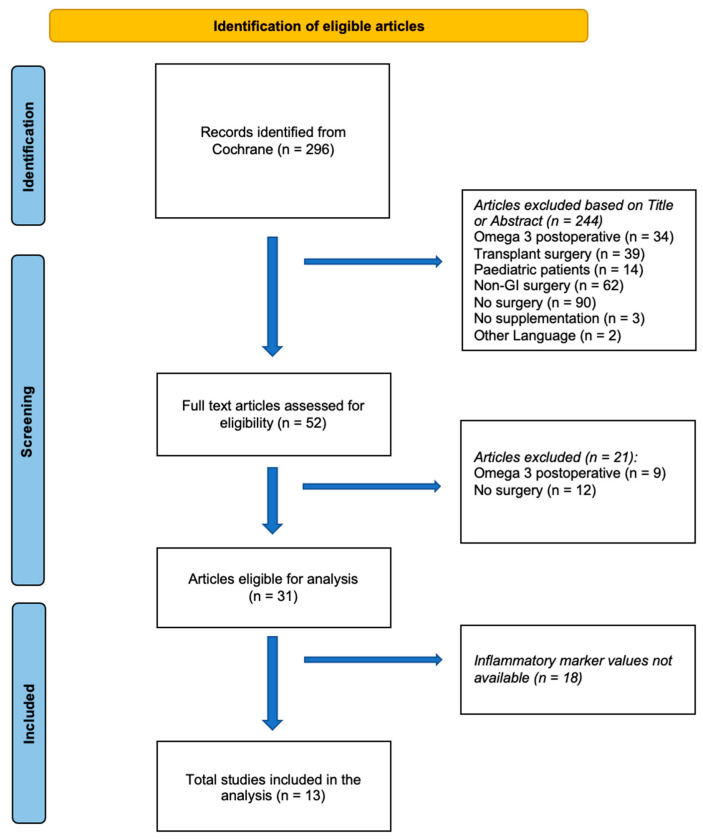
Flow chart demonstrating the process of study selection (PRISMA Chart).

A total of 296 articles were identified in Cochrane after applying our search criteria. Among these, 244 articles were excluded based on their title or abstract. Details of the exclusion process can be found in Figure 1.

Out of the original 31 studies that met our inclusion criteria, 18 had to be excluded due to lack of inflammatory marker values. Therefore, only 13 articles including n = 950 patients were incorporated into the subsequent analysis (Figure 1). Data from the included studies are in Table 1.

Our decision to analyze IL-6, CRP, and WBC was based on their prevalence as the most commonly observed markers in the included trials.

IL-6 data were extracted from 10 studies, CRP values from 7, and WBC data from 3 publications. The first respective postoperative value was compared among groups in each study. With respect to clinically relevant outcome measures, hospital LOS were reported consistently, and the corresponding data could be extracted from five trials, which were included in this analysis.

The mean age of patients for the control and intervention groups ranged from 46.4 to 69.0 years and from 45.5 to 67.5 years, respectively. The dosage of omega-3 FAs used ranged from 2 g/day to 6.5 g/day. The administration of omega-3 FAs was through the enteral route in 11 studies and intravenous in 2.

Time of initiation ranged from 10 days before the planned procedure, at the earliest, to one day preoperatively, at the latest. Omega-3 administration was carried out until the day of the operation, at the earliest, and until 21 days postoperation, at the latest.

### 3.1. Interleukin 6

**Figure 2:**
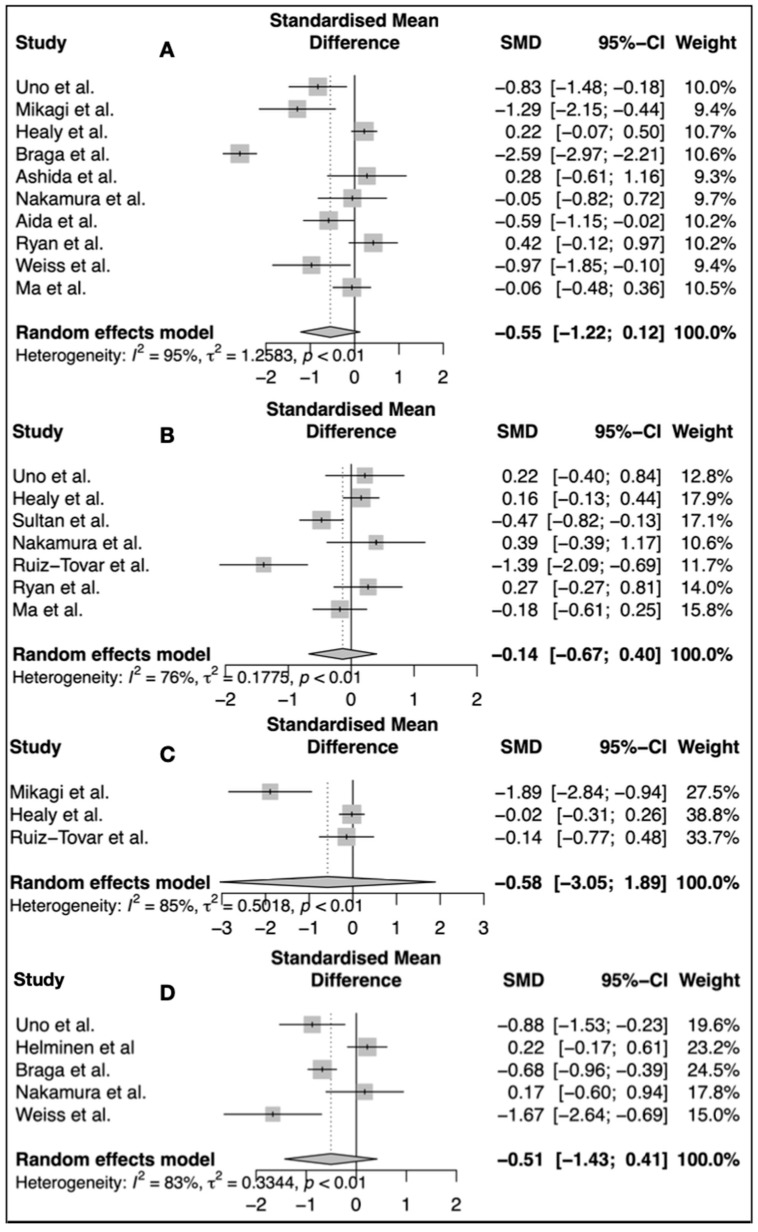
Effects of omega-3 Fatty acids on post operative inflammatory response: (**A**) IL-6 [30,31,32,33,34,35,36,37,38,39] (**B**) CRP [30,32,36,38,39,40,41] (**C**) WBC [31,32,41] (**D**) Length of Hospital Stay (LOS) [30,33,37,39,42].

IL-6 data were extracted from 10 studies (n = 689) [30,31,32,33,34,35,36,37,38,39]. The unit used for IL-6 was pg/mL. The first postoperative IL-6 values were compared among groups in each study. As demonstrated in Figure 2, IL-6 levels were lower in the omega-3 group SMD: −0.55 [−1,22; 0.12], *p* = 0.10. However, this difference did not reach statistical significance (Figure 2A).

### 3.2. C-Reactive Protein

CRP data from seven studies were extracted and analyzed (n = 605) [30,32,36,38,39,40,41]. The unit used for CRP was mg/dL. The first postoperative CRP values were compared among groups in each study. CRP values tended to be lower in the omega-3 group SMD: −0.14 [−0.67; 0.40] *p* = 0.55. However, there was no statistically significant difference between groups (Figure 2B).

The CRP data from Helminen et al. were only available as median values and, therefore, were not included in the analysis [42].

### 3.3. White Blood Count

Only three studies (n = 257) reported WBC counts and were included in the corresponding analysis [31,32,41]. The unit used for WBC was WBC/mL. The first postoperative WBC count was included and compared among the groups. Similarly to IL-6 and CPR, WBC counts also tended to be lower in the omega-3 group SMD: −0.58 [−3.05; 1.89]. Similarly to IL-6 and CRP, there was no statistically significant difference *p* = 0.42 (Figure 2C).

### 3.4. Length of Hospital Stay

Length of hospital stay (LOS) was analyzed using data from five trials (n = 389) [30,33,37,39,42], since this was the most common reported clinical variable. As shown in Figure 2D, the hospital LOS was also shorter in patients treated with the omega-3; however, it did not reach statistical significance SMD: −0.51 [−1.43; 0.41] *p* = 0.20.

**Table 1:** Summary data of studies included in the meta-analysis: *: published IL-6 Data in median. ** published age data in median, *** published LOS data in median, **** Omega 3 dose was calculated using the mean weight of the group [30,31,32,33,34,35,36,37,38,39,40,41,42].

No.	Author	Country	Surgical Procedure	Sample Size (Control/Intervention)	Definition of Control	Age (Control/Intervention)	Dosis of Omega 3 g/d	Route of Administratio	Time of Initiation (Day 0: Operation)	Duration of Therapy	LOS Control/Intervention
1	Uno et al. [30]	Japan	Major hepatobiliary resection	40 (20/20)	No supplementation	66.4/65.5	2.6	Enteral	−5	−5 d–>0 d	Control: 53.9 ± 5 Intervention: 36.9 ± 3.3
2	Mikagi et al. [31]	Japan	Segmentectomy or extensive hepatectomy	26 (13/13)	No supplementation	61.5/67.5	3.1	Enteral	−5	−5 d–>0 d	Control: 14.5 Intervention: 16.3
3 *	Helminen et al. [42]	Finland	Gastrointestinal cancer operations	100 (50/50)	No supplementation	63/58	3	Enteral	−5	−5 d–>+5 d	Control: 9 ± 5 Intervention: 10 ± 4
4 **	Healy et al. [32]	Ireland	Esophagectomy	191 (94/97)	No supplementation	62/62	2.2	Enteral	−5	−1 d–>+30 d	No mentioned data
5	Braga et al. [33]	Italy	Colorectal cancer surgery	200 (100/100)	50 no supplementation 50 isocaloric, isonitrogenous nutrition	No supplementation 62.6: control. 61.8 only preOp Omega 3 FA 63: Pre and postOp 60.5	3.3	Enteral	−5	preOp: −5 d–>0 d Pre + PostOp: −5 d–>+8 d	No supp.: 12.2 ± 3.9 Control Supp.: 12.0 ± 4.5 preOp Omega 3: 9.5 ± 2.9 pre and postOp: 9.8 ± 3.1
6	Ashida et al. [34]	Japan	Pancreatoduodenectomy	20 (9/11)	Isocaloric isonitrogenous nutrition	69/64	2	Enteral	−7	−7 d–>0 d	No mentioned data
7 ***	Sultan et al. [40]	UK	Esophagogastric cancer surgery	195 (129/66)	66 no supplementation 63 Standard enteral nutrition without immunonutrients	No supplementation: 66 Enteral nutrition: 60 Intervention: 67	4.92	Enteral	−7	−7 d–>+7 d	Conventional: 16 (11–34) Control: 16 (11–116) Intervention: 18 (4–141)
8	Nakamura et al. [39]	Japan	Bile duct cancer/pancreatic cancer, gastric cancer, esophageal cancer	26 (14/12)	No supplementation	60.75/65	4	Enteral	−5	−5 d–>0 d	Control: 46.1 ± 15 Intervention: 49.0 ± 18.3
9	Aida et al. [35]	Japan	Pancreatoduodenectomy	50 (25/25)	No supplementation	65.1/66.4	4	Enteral	−5	−5 d–>0 d	No mentioned data
10 ***	Ruiz-Tovar et al. [41]	Spain	Roux-en-Y gastric bypass	40 (20/20)	balanced energy high-protein formula	46.4/45.5	4.26	Enteral	−10	−10 d–>0 d	Control: 2 Intervention: 2
11	Ryan et al. [36]	Ireland	Esophagectomy	53 (25/28)	isocaloric isonitrogenous standard nutritional feed	65.7/62	2.2	Enteral	−5	−5 d–>+21 d	No mentioned data
12	Weiss et al. [37]	DE	Extended abdominal surgery	23 (11/12)	Parenteral nutrition with glucose, amino acids and fat	61.6/57.4	4	Intravenous	−1	−1 d–>+5 d	Control: 23.5 Intervention: 17.8
13 ****	Ma et al. [38]	Taiwan	Gastrointestinal cancer operations	86 (41/45)	Soybean oil and medium-chain triglycerides	62.85/61.55	6.5 g mean weight	Intravenous	−1	−1 d–>+7 d	No mentioned data

## 4. Discussion

We identified 13 trials, adding up to 950 patients, that were included in this meta-analysis. Intervention was defined as the preoperative initiation of omega-3 FA administration. Studies investigating only postoperative administration were not included, since this administration might have lower or delayed consequences on inflammatory mediators compared to presurgical initiation. Conventional strategy of care and nutritional supplementation not containing omega-3 FA were considered as control. 

Our findings demonstrated only small effects toward reduced inflammatory mediators as well as on the length of hospital stay in patients who received omega-3 fatty acid supplementation prior to surgery. None of these results reached statistical significance, a fact which may be due to insufficient evidence gathered from rather small cohorts of patients. 

Patients undergoing abdominal surgery frequently experience an ischemia/reperfusion injury and surgical trauma, both triggering the release of proinflammatory cytokines including interleukin-1 and tumor necrosis factor-α (TNF-α), which ultimately leads to a systemic inflammatory response [1]. These, in turn, sequentially activate the release of other cytokines, including IL-6 [1,43], which further stimulates acute phase proteins including CRP [43], the latter being recognized as an important and reliable inflammatory marker by current international nutrition guidelines [44]. 

Omega-3 fatty acids possess potent anti-inflammatory properties that have been demonstrated in several experimental studies and clinical trials examining pre-emptive omega-3 supplementation’s impact on inflammatory response [17,45,46]. However, these anti-inflammatory effects have not been conclusively investigated in the perioperative setting.

We performed this meta-analysis based on the premise that beneficial effects of preoperative omega-3 FAs administration would attenuate the inflammatory response triggered by surgery in the early postsurgical stage. To our knowledge, there is no data supporting this hypothesis regarding the postsurgical inflammatory response. However, an experimental study by Yan et al. found that docosahexaenoic acid (DHA) inhibited caspase-1 and interleukin-1β (IL-1β) activity in bone-marrow-derived macrophages (BMDMs) pretreated with lipopolysaccharide (LPS) and decreased the secretion of interleukin-18 (IL-18), another acute phase interleukin. Other omega-3 FAs, eicosapentaenoic acid (EPA), and alpha-linolenic acid (ALA) showed similar effects [45]. It also showed that DHA did not affect the lipopolysaccharide (LPS)-induced priming for activation of NF-kB and TNF-α when added after the LPS treatment; it did, however, inhibit this process when applied before the LPS treatment, suggesting that DHA has protective effects as a preemptive treatment. Furthermore, DHA was found to inhibit NLRP3 and NLRP1b inflammasome activation triggered by a variety of agonists, implying a broad inhibitory effect and, consequently, inhibiting the IL-1β production. This indicates their potential role in modulating immune responses. This, among other studies, led us to our hypothesis that administering omega-3 fatty acids prior to surgical trauma might be more effective in reducing the inflammatory response than administering them after surgical trauma.

Several meta-analyses showed that the administration of omega-3 FAs during the perioperative course reduced the length of stay in the intensive care unit and the length of hospital stay, as well as reducing the risk of postoperative infections [9,47,48,49,50]. A meta-analysis by Wei et al., consisting of six trials, reported a significant reduction in infections and length of hospital stay after postoperative administration of emulsions containing fish oil. However, it is important to note that Wei et al. used the random model analyzing heterogeneous surgical procedures including cancer, abdominal, and vascular surgeries, as well as different administration strategies [51]. In 2017, Bae et al. demonstrated in a meta-analysis that intravenous omega-3 FAs are beneficial with respect to infectious morbidity and length of hospital stay when compared to soybean oil emulsions [47]. 

Furthermore, a recent meta-analysis by Lu et al. revealed that the implementation of perioperative omega-3 in gastrointestinal cancer patients undergoing gastrointestinal cancer surgery markedly diminished the inflammatory response while concurrently reducing the length of hospital stay. This meta-analysis included 10 trials comprising 663 patients and did not differentiate between the omega-3 FAs that were administered either preoperatively or postoperatively [52].

Similarly, Xiao et al. conducted a meta-analysis aimed at investigating the potential impact of omega-3 supplementation on patients subjected to liver surgery. Their investigation yielded a reduction in the incidence of postoperative infections, although they did not observe a significant impact on the incidence rate of other complications [53].

However, a notable distinction should be made here. Xiao and colleagues’ analysis focused on the clinical infections, meaning that, unlike our research, they did not analyze the inflammatory mediators. Interestingly, their analysis also found that a prolonged regimen of supplementation, both pre- and post-surgery, exhibited a higher degree of effectiveness in mitigating complication rates when compared to a singular dose regimen.

A recent umbrella review conducted by Slim and colleagues evaluated the role of immunonutrition (IN) (nutrition containing, among others, omega-3 FAs, arginine, glutamine, and nucleotides) in perioperative settings, revealing its significant potential in reducing postoperative complications, particularly infectious complications, by nearly 50%, regardless of its timing of initiation [54]. This review synthesized the findings of 20 meta-analyses, which in total comprised 133 randomized trials focusing on the use of IN in abdominal surgery. Despite the comprehensive approach taken by this umbrella review, it failed to provide convincing evidence regarding the effectiveness of IN in relation to the patient’s condition, the specific components of IN, and the optimal timing of IN use. This was mainly due to the generally low quality of the existing data [54].

While these previous reports investigated perioperative administration, our meta-analysis aimed to investigate the impact of preoperative omega-3 nutrition on the postoperative inflammatory response. 

With this reported emerging interest of omega-3 FAs, concerns have increased regarding potential bleeding complications during major surgeries due to their consumption [55]. It has been hypothesized that eicosapentaenoic acid (EPA) and docosahexaenoic acid (DHA) vie with arachidonic acid in the platelet membrane, consequently resulting in a diminished production of arachidonic-acid-derived prothrombotic metabolites. This might also lead to an augmented synthesis of antithrombotic EPA metabolites, which may contribute to diminished platelet aggregation [55,56]. This issue was investigated by a secondary analysis of the OPERA Trial, which was a multinational, placebo-controlled randomized trial evaluating the impact of fish oil on the incidence of atrial fibrillation post cardiac surgery [57]. This analysis revealed no increase in perioperative bleeding incidence following fish oil supplementation; instead, a significant decrease in the number of blood transfusion was observed. Furthermore, higher plasma levels of omega-3 FAs were correlated with a reduced risk of bleeding [55]. To our knowledge, no large randomized trials have demonstrated a higher bleeding risk after consumption of omega-3 FAs.

Although omega-3 fatty acids have been the subject of extensive research, there remain certain challenges regarding their administration, with dosing being an important point of concern. The inconsistency in the dosing regimens observed across different studies intensifies the need for further investigations. As expressed by Nishizaki et al., it is crucial that research takes into consideration the variations in baseline EPA/AA ratios and the possible impact of the EPA/AA threshold on the outcome of future studies [58].

Another important aspect would be the duration of omega-3 FAs administration. As it stands, we do not yet have a clear understanding of what the optimal duration for supplementation is, which might vary depending on the specific aims of the supplementation. Evidence from studies such as Katan et al. and Browning et al. suggests that the process of omega-3 FAs incorporation into the tissue is rather slow. This implies that it could take several months to reach a steady state following the start of supplementation [59,60]. 

When interpreting our findings, it is important to acknowledge the existence of several limitations, including the heterogeneity of disease and surgery among the abdominal surgery trials included. In addition, the diversity of dosing regimens, administration routs, timing of initiation, combination with other immunonutrients, and duration of omega-3 FAs administration must be considered as sources of heterogeneity that may have significantly influenced the obtained findings. Different diseases and surgical interventions may instigate varying inflammatory responses, potentially requiring a diverse range of omega-3 fatty acid dosages for optimal attenuation of these immune reactions. Additionally, the contrasting time of initiation of the supplementation could modulate its effectiveness, either amplifying or diminishing the impact on the inflammatory response. It is also crucial to underscore the variance in the EPA/DHA ratio across the trials included in this analysis. This inconsistency could be perceived as a limitation.

Despite the fact that hospital length of stay is influenced by multiple factors, including non-clinical ones, it was included as the sole clinical parameter in our analysis, as it has been included in previous studies. The primary endpoint of this investigation was the postoperative inflammatory markers; hence, the length of stay data were solely acquired from those studies which had reported the inflammatory markers.

A further limitation to our investigation is the broad definition of the control group, which was a direct consequence of the limited availability of published data and of the small cohort sizes in the studies included. Consequently, our analysis was unable to differentiate between conventional treatment (no administration) and treatments with non-omega-3 nutrition. Moreover, our investigation encountered a number of studies that presented incomplete data, and, despite attempts to obtain original research data from the corresponding authors, this information remained unavailable. It is important to note and account for this limitation when assessing the quality of future studies in this area. The lack of available raw data from primary studies and the unresponsiveness of authors represent a major issue that should be considered by editors and journals when publishing future studies.

In order to address this issue in our study, we utilized the WebPlotDigitizer application to extract the necessary data. While this approach has been successfully used in previous studies, it is important to recognize that the accuracy of the obtained data might be somewhat inferior to that of the original source material [61].

In conclusion, given the current ambiguous state of available data, there is a significant need for further methodologically rigorous investigations such as randomized controlled trials to investigate the causality between omega-3 supplementation and potential beneficial effects on the postoperative inflammatory response. These investigations should compare omega-3 administration to other supplementations, as well as to the conventional practice that does not involve supplementation. The establishment of optimal dosing regimens with the optimal EPA/DHA ratio, route of administration, timing of initiation, and duration of therapy will require thorough comparative studies to determine the most beneficial supplementation protocol. Potentially, the measurement of EPA/AA ratio may play a role in such an investigation. In this analysis, it would be highly interesting to incorporate additional inflammatory markers such as TNF alpha, Interleukin-1, and Procalcitonin as well as clinical parameters such as duration of hospital stay, incidence of surgical site infection, and postoperative bleeding.

Comparative studies would also be important to elucidate the optimal content and timing for omega-3 supplementation. Such studies should include a cohort that receives only preoperative supplementation, a second cohort that begins supplementation preoperatively and continues the treatment postoperatively, and a third cohort that initiates supplementation postoperatively. Such a study design may provide vital insights into the effects of this supplementation, ultimately facilitating the identification of the most beneficial timing to optimize the impact on the postoperative inflammatory response.

## Data Availability

Available upon request.
